# Unfolding dermatological spectrum of Still’s disease: a cohort study from the International AIDA Network Still’s Disease Registry

**DOI:** 10.1093/rheumatology/keaf512

**Published:** 2025-09-30

**Authors:** Laura Calabrese, Martina D’Onghia, Alessandra Cartocci, Andrea Hinojosa-Azaola, Jiram Torres-Ruiz, Giuseppe Lopalco, Jessica Sbalchiero, Valeria Caggiano, Henrique A Mayrink Giardini, Ibrahim A Almaghlouth, Piero Ruscitti, Ilenia Di Cola, Petros P Sfikakis, Katerina Laskari, Paolo Sfriso, Lorenzo Dagna, Corrado Campochiaro, Abdurrahman Tufan, Hamit Kucuk, Riza Can Kardas, Abdulsamet Erden, Gaafar Ragab, Mohamed Tharwat Hegazy, Ahmed Hatem Laymouna, Luca Navarini, Onorina Berardicurti, Francesco Ciccia, Daniela Iacono, Flavia Riccio, Lampros Fotis, Haner Direskeneli, Joanna Makowska, Annamaria Iagnocco, Alessandro Conforti, Donato Rigante, Maissa Thabet, Florenzo Iannone, Daniele Domanico, Marcello Govoni, Maria Cristina Maggio, Emanuela Del Giudice, Francesco La Torre, Ezgi D Batu, Seza Ozen, Carla Gaggiano, Eduardo Martín-Nares, Guillermo Arturo Guaracha-Basañez, Anastasios Karamanakos, Alberto Lo Gullo, Benedetta Monosi, Elena Bartoloni, José Hernández-Rodríguez, Verónica Gómez-Caverzaschi, Giacomo Emmi, Sukran Erten, Francesco Carubbi, Maria Francesca Gicchino, Amato De Paulis, Giovanni Conti, Benson Ogunjimi, Ewa Wiesik-Szewczyk, Anna Nowakowska-Płaza, Ombretta Viapiana, Piercarlo Sarzi-Puttini, Samar Tharwat, Francesca Crisafulli, Paola Parronchi, Antonio Gidaro, Ludovico De Stefano, Luciana Breda, Lidia La Barbera, Giuliana Guggino, Albero Balistreri, Claudia Fabiani, Pietro Rubegni, Bruno Frediani, Roberto Giacomelli, Luca Cantarini, Antonio Vitale, International AIDA (AutoInflammatory Diseases Alliance) Network and the Autoinflammatory Diseases Working Group of the Italian Society of Rheumatology (SIR), Jessica Sbalchiero, Valeria Caggiano, Piero Ruscitti, Ilenia Di Cola, Paolo Sfriso, Lorenzo Dagna, Corrado Campochiaro, Francesco Ciccia, Dianela Iacono, Flavia Riccio, Annamaria Iagnocco, Florenzo Iannone, Giuseppe Lopalco, Marcello Govoni, Carla Gaggiano, Elena Bartoloni, Giacomo Emmi, Ombretta Viapiana, Francesca Crisafulli, Lidia La Barbera, Carla Guggino, Claudia Fabiani, Bruno Frediani, Roberto Giacomelli, Luca Cantarini, Antonio Vitale

**Affiliations:** Dermatology Unit, Department of Medical, Surgical and Neurological Sciences, University of Siena, Siena, Italy; Institute of Dermatology, Catholic University of the Sacred Heart, Rome, Italy; Azienda Ospedaliero-Universitaria Senese [European Reference Network (ERN) for Rare Immunodeficiency, Autoinflammatory and Autoimmune Diseases (RITA) Center], Siena, Italy; Dermatology Unit, Department of Medical, Surgical and Neurological Sciences, University of Siena, Siena, Italy; Azienda Ospedaliero-Universitaria Senese [European Reference Network (ERN) for Rare Immunodeficiency, Autoinflammatory and Autoimmune Diseases (RITA) Center], Siena, Italy; Dermatology Unit, Department of Medical, Surgical and Neurological Sciences, University of Siena, Siena, Italy; Azienda Ospedaliero-Universitaria Senese [European Reference Network (ERN) for Rare Immunodeficiency, Autoinflammatory and Autoimmune Diseases (RITA) Center], Siena, Italy; Department of Immunology and Rheumatology, Instituto Nacional de Ciencias Médicas y Nutrición Salvador Zubirán, Mexico City, Mexico; Department of Immunology and Rheumatology, Instituto Nacional de Ciencias Médicas y Nutrición Salvador Zubirán, Mexico City, Mexico; Department of Precision and Regenerative Medicine and Ionian Area (DiMePRe-J) Policlinic Hospital, University of Bari, Bari, Italy; Azienda Ospedaliero-Universitaria Senese [European Reference Network (ERN) for Rare Immunodeficiency, Autoinflammatory and Autoimmune Diseases (RITA) Center], Siena, Italy; Department of Medical Sciences, Surgery and Neurosciences, Research Center of Systemic Autoinflammatory Diseases and Behçet’s Disease Clinic, University of Siena, Siena, Italy; Azienda Ospedaliero-Universitaria Senese [European Reference Network (ERN) for Rare Immunodeficiency, Autoinflammatory and Autoimmune Diseases (RITA) Center], Siena, Italy; Department of Medical Sciences, Surgery and Neurosciences, Research Center of Systemic Autoinflammatory Diseases and Behçet’s Disease Clinic, University of Siena, Siena, Italy; Rheumatology Division, Faculdade de Medicina, Hospital das Clínicas, Universidade de São Paulo, São Paulo, Brazil; Rheumatology Unit, Department of Medicine, College of Medicine, King Saud University, Riyadh, Saudi Arabia; College of Medicine Research Center, College of Medicine, King Saud University, Riyadh, Saudi Arabia; Department of Biotechnological and Applied Clinical Sciences, University of L'Aquila, L'Aquila, Italy; Department of Biotechnological and Applied Clinical Sciences, University of L'Aquila, L'Aquila, Italy; Joint Academic Rheumatology Program, Medical School, National and Kapodistrian University of Athens, Athens, Greece; Rheumatology Unit, 1st Dept. of Propaedeutic Internal Medicine, National & Kapodistrian University of Athens Medical School, Athens, Greece; Rheumatology Unit, Department of Medicine, University of Padua, [European Reference Network (ERN) for Rare Immunodeficiency, Autoinflammatory and Autoimmune Diseases (RITA) Center], Padua, Italy; Faculty of Medicine, Università Vita-Salute San Raffaele, Milan, Italy; Unit of Immunology, Rheumatology, Allergy and Rare Diseases, IRCCS Ospedale San Raffaele, Milan, Italy; Faculty of Medicine, Università Vita-Salute San Raffaele, Milan, Italy; Unit of Immunology, Rheumatology, Allergy and Rare Diseases, IRCCS Ospedale San Raffaele, Milan, Italy; Department of Internal Medicine, Division of Rheumatology, Gazi University Hospital, Ankara, Turkey; Department of Internal Medicine, Division of Rheumatology, Gazi University Hospital, Ankara, Turkey; Department of Internal Medicine, Division of Rheumatology, Gazi University Hospital, Ankara, Turkey; Department of Internal Medicine, Division of Rheumatology, Gazi University Hospital, Ankara, Turkey; Internal Medicine Department, Rheumatology and Clinical Immunology Unit, Faculty of Medicine, Cairo University, Giza, Egypt; Faculty of Medicine, Newgiza University, Giza, Egypt; Internal Medicine Department, Rheumatology and Clinical Immunology Unit, Faculty of Medicine, Cairo University, Giza, Egypt; Faculty of Medicine, Newgiza University, Giza, Egypt; Internal Medicine Department, Rheumatology and Clinical Immunology Unit, Faculty of Medicine, Cairo University, Giza, Egypt; Clinical and research section of Rheumatology and Clinical Immunology, Fondazione Policlinico Campus Bio-Medico, Rome, Italy; Department of Medicine, University of Rome “Campus Biomedico” School of Medicine, Rheumatology, Immunology and Clinical Medicine Unit, Rome, Italy; Clinical and research section of Rheumatology and Clinical Immunology, Fondazione Policlinico Campus Bio-Medico, Rome, Italy; Department of Medicine, University of Rome “Campus Biomedico” School of Medicine, Rheumatology, Immunology and Clinical Medicine Unit, Rome, Italy; Department of Precision Medicine, University of Campania “Luigi Vanvitelli”, Naples, Italy; Department of Precision Medicine, University of Campania “Luigi Vanvitelli”, Naples, Italy; Department of Precision Medicine, University of Campania “Luigi Vanvitelli”, Naples, Italy; Department of Pediatrics, Attikon General Hospital, National and Kapodistrian University of Athens, Athens, Greece; Department of Internal Medicine, Division of Rheumatology, Marmara University, Faculty of Medic, Istanbul, Turkey; Department of Rheumatology, Medical University of Lodz, Lodz, Poland; Academic Rheumatology Centre, Dipartimento Scienze Cliniche e Biologiche, Università degli Studi di Torino, Torino, Italy; U.O. Medicina Generale, Ospedale San Paolo di Civitavecchia, ASL Roma 4, Civitavecchia, Rome, Italy; Department of Life Sciences and Public Health, Fondazione Policlinico Universitario A. Gemelli IRCCS, Rome, Italy; Periodic Fever Research Center, Università Cattolica Sacro Cuore, Rome, Italy; Internal Medicine Department, Farhat Hached University Hospital, Sousse, Tunisia; Faculty of Medicine of Sousse, University of Sousse, Sousse, Tunisia; Department of Precision and Regenerative Medicine and Ionian Area (DiMePRe-J) Policlinic Hospital, University of Bari, Bari, Italy; Department of Precision and Regenerative Medicine and Ionian Area (DiMePRe-J) Policlinic Hospital, University of Bari, Bari, Italy; Rheumatology Unit, Department of Medical Sciences, Azienda Ospedaliero-Universitaria S. Anna-Ferrara, University of Ferrara, Ferrara, Italy; University Department of Health Promotion, Mother and Child Care, Internal Medicine and Medical Specialties (PROMISE) “G. D'Alessandro”, University of Palermo, Palermo, Italy; Pediatric and Neonatology Unit, Department of Maternal Infantile and Urological Sciences, Sapienza University of Rome, Latina, Italy; Department of Pediatrics, Pediatric Rheumatology Center, Giovanni XXIII Pediatric Hospital, University of Bari, Bari, Italy; Department of Pediatric Rheumatology, Faculty of Medicine, Hacettepe University, Ankara, Turkey; Department of Pediatric Rheumatology, Faculty of Medicine, Hacettepe University, Ankara, Turkey; Azienda Ospedaliero-Universitaria Senese [European Reference Network (ERN) for Rare Immunodeficiency, Autoinflammatory and Autoimmune Diseases (RITA) Center], Siena, Italy; Department of Medical Sciences, Surgery and Neurosciences, Research Center of Systemic Autoinflammatory Diseases and Behçet’s Disease Clinic, University of Siena, Siena, Italy; Department of Immunology and Rheumatology, Instituto Nacional de Ciencias Médicas y Nutrición Salvador Zubirán, Mexico City, Mexico; Department of Immunology and Rheumatology, Instituto Nacional de Ciencias Médicas y Nutrición Salvador Zubirán, Mexico City, Mexico; Department of Rheumatology, “Evangelismos” General Hospital, Athens, Greece; UOSD Reumatologia, ARNAS Garibaldi, Catania, Italy; Rheumatology, Allergology and Clinical Immunology, Fondazione Policlinico Tor Vergata, University of Rome Tor Vergata, Rome, Italy; Section of Rheumatology, Department of Medicine and Surgery, University of Perugia, Perugia, Italy; Clinical Unit of Autoinflammatory Diseases, Department of Autoimmune Diseases, Institut d‘Investigacions Biomèdiques August Pi I Sunyer (IDIBAPS), Hospital Clínic of Barcelona [European Reference Network (ERN) for Rare Immunodeficiency, Autoinflammatory and Autoimmune Diseases (RITA) Center], University of Barcelona, Barcelona, Spain; Clinical Unit of Autoinflammatory Diseases, Department of Autoimmune Diseases, Institut d‘Investigacions Biomèdiques August Pi I Sunyer (IDIBAPS), Hospital Clínic of Barcelona [European Reference Network (ERN) for Rare Immunodeficiency, Autoinflammatory and Autoimmune Diseases (RITA) Center], University of Barcelona, Barcelona, Spain; Department of Medical, Surgical and Health Sciences, University of Trieste, Trieste, Italy; Clinical Medicine and Rheumatology Unit, Cattinara University Hospital, Trieste, Italy; Centre for Inflammatory Diseases, Monash University Department of Medicine, Monash Medical Centre, Melbourne, Australia; Department of Rheumatology, Faculty of Medicine Ankara City Hospital, Ankara Yıldırım Beyazıt University, Ankara, Turkey; Department of Life, Health & Environmental Sciences and Internal Medicine and Nephrology Unit, Department of Medicine, University of L'Aquila and ASL Avezzano-Sulmona-L'Aquila, San Salvatore Hospital, L'Aquila, Italy; Department of Woman, Child and of General and Specialized Surgery, University of Campania Luigi Vanvitelli, Napoli, Italy; Department of Translational Medical Sciences, Section of Clinical Immunology, University of Naples Federico II, Naples, Italy; Center for Basic and Clinical Immunology Research (CISI), WAO Center of Excellence, University of Naples Federico II, Naples, Italy; Pediatric Nephrology and Rheumatology Unit, Azienda Ospedaliera Universitaria (AOU), “G. Martino”, Messina, Italy; “Antwerp Center for Translational Immunology and Virology, Vaccine and Infectious Disease Institute, University of Antwerp, Antwerp, Belgium; Antwerp Unit for Data Analysis and Computation in Immunology and Sequencing, University of Antwerp, Antwerp, Belgium; Department of Paediatrics, Antwerp University Hospital, Antwerp, Belgium; Center for Health Economics Research and Modeling Infectious Diseases, Vaccine and Infectious Disease Institute, University of Antwerp, Antwerp, Belgium; Department of Internal Medicine, Pneumonology, Allergology, Clinical Immunology and Rare Diseases, Military Institute of Medicine National Research Institute, Warsaw, Poland; Department of Internal Medicine, Pneumonology, Allergology, Clinical Immunology and Rare Diseases, Military Institute of Medicine National Research Institute, Warsaw, Poland; Rheumatology Unit, Department of Medicine, University and Azienda Ospedaliera Universitaria Integrata of Verona, Verona, Italy; Rheumatology Unit, IRCCS Ospedale Galeazzi-S. Ambrogio, Università degli Studi di Milano, Milan, Italy; Rheumatology and Immunology Unit, Internal Medicine Department, Mansoura University, Mansoura, Egypt; Department of Internal Medicine, Faculty of Medicine, Horus University, New Damietta, Egypt; Rheumatology and Clinical Immunology, Spedali Civili and Department of Clinical and Experimental Sciences, University of Brescia, [European Reference Network (ERN) for Rare Immunodeficiency, Autoinflammatory and Autoimmune Diseases (RITA) Center], Brescia, Italy; Department of Experimental and Clinical Medicine, University of Florence, Florence, Italy; Department of Biomedical and Clinical Sciences Luigi Sacco, Luigi Sacco Hospital, University of Milan, Milan, Italy; Rheumatology Department, Istituto di ricovero e cura a carattere scientifico Policlinico S. Matteo Fondazione, University of Pavia, Pavia, Italy; Department of Paediatrics, University of Chieti-Pescara, Chieti, Italy; Rheumatology Section, Department of Health Promotion, Mother and Child Care, Internal Medicine and Medical Specialties, University Hospital P. Giaccone, Palermo, Italy; Rheumatology Section, Department of Health Promotion, Mother and Child Care, Internal Medicine and Medical Specialties, University Hospital P. Giaccone, Palermo, Italy; Azienda Ospedaliero-Universitaria Senese [European Reference Network (ERN) for Rare Immunodeficiency, Autoinflammatory and Autoimmune Diseases (RITA) Center], Siena, Italy; Bioengineering and Biomedical Data Science Lab, Department of Medical Biotechnologies, University of Siena, Siena, Italy; Azienda Ospedaliero-Universitaria Senese [European Reference Network (ERN) for Rare Immunodeficiency, Autoinflammatory and Autoimmune Diseases (RITA) Center], Siena, Italy; Ophthalmology Unit, Department of Medicine, Surgery and Neurosciences, University of Siena, Siena, Italy; Dermatology Unit, Department of Medical, Surgical and Neurological Sciences, University of Siena, Siena, Italy; Azienda Ospedaliero-Universitaria Senese [European Reference Network (ERN) for Rare Immunodeficiency, Autoinflammatory and Autoimmune Diseases (RITA) Center], Siena, Italy; Azienda Ospedaliero-Universitaria Senese [European Reference Network (ERN) for Rare Immunodeficiency, Autoinflammatory and Autoimmune Diseases (RITA) Center], Siena, Italy; Department of Medical Sciences, Surgery and Neurosciences, Research Center of Systemic Autoinflammatory Diseases and Behçet’s Disease Clinic, University of Siena, Siena, Italy; Clinical and research section of Rheumatology and Clinical Immunology, Fondazione Policlinico Campus Bio-Medico, Rome, Italy; Department of Medicine, University of Rome “Campus Biomedico” School of Medicine, Rheumatology, Immunology and Clinical Medicine Unit, Rome, Italy; Azienda Ospedaliero-Universitaria Senese [European Reference Network (ERN) for Rare Immunodeficiency, Autoinflammatory and Autoimmune Diseases (RITA) Center], Siena, Italy; Department of Medical Sciences, Surgery and Neurosciences, Research Center of Systemic Autoinflammatory Diseases and Behçet’s Disease Clinic, University of Siena, Siena, Italy; Azienda Ospedaliero-Universitaria Senese [European Reference Network (ERN) for Rare Immunodeficiency, Autoinflammatory and Autoimmune Diseases (RITA) Center], Siena, Italy; Department of Medical Sciences, Surgery and Neurosciences, Research Center of Systemic Autoinflammatory Diseases and Behçet’s Disease Clinic, University of Siena, Siena, Italy

**Keywords:** registry, AIDA network, Still’s disease, skin lesions, rare diseases, autoinflammatory diseases, systemic juvenile idiopathic arthritis

## Abstract

**Objectives:**

To investigate cutaneous manifestations in Still’s disease patients, evaluating any correlation with ethnic origin, age at disease onset, disease patterns, occurrence of macrophage activation syndrome (MAS) and systemic activity scores.

**Methods:**

Data were retrospectively drawn from the International AutoInflammatory Disease Alliance (AIDA) Network Registry dedicated to Still’s disease.

**Results:**

A total of 518 patients (41.3% males) were enrolled. Salmon-coloured evanescent skin rash (*n* = 304, 63.9%), macules (*n* = 40, 7.7%), urticarial eruptions (*n* = 31, 5.9%), erythema (*n* = 27, 5.2%) and persistent pruritic papules and plaques (PPPP) (*n* = 25, 4.8%) accounted for the most frequent skin manifestations observed in Still’s disease. Overall, atypical skin rash were described in 110 (21.2%) patients. Salmon-coloured evanescent skin rash and pruritus were more common among patients aged <16 years compared with patients aged 16–60 (*P* = 0.002 and *P* = 0.008, respectively). Pruritus was significantly more frequent among White than among Arab patients (*P* = 0.008) and in polycyclic vs monocyclic course (*P* = 0.049). Hispanics showed a significantly higher rate of atypical skin manifestations compared with Arabs (*P* = 0.036) and White (*P* = 0.036). Also, macules were more frequent among Hispanics than White (*P* = 0.027), while PPPP was more frequent among Hispanics than Arabs (*P* = 0.023) and White (*P* = 0.002). Salmon-coloured evanescent skin rash was significantly more frequent among patients with a systemic activity score ≥7 (*P* < 0.001).

**Conclusion:**

The present study enhances dermatologists’ awareness of the diverse cutaneous lesions that may represent heterogeneous manifestations of Still’s disease, shedding new light on the difference related to the age at disease onset, the patients’ ethnic origin and the severity of the disease.

Rheumatology key messages

- Cutaneous manifestations in Still’s disease vary significantly by age, ethnicity and disease severity.
- Atypical skin lesions are more frequent among Hispanic patients and older age groups.
- Typical salmon-coloured rash correlates with higher systemic disease activity.

## Introduction

Still’s disease is a systemic autoinflammatory disease more frequently affecting children and young adults [1]. Fever, arthralgia, arthritis, lymphadenopathy, splenomegaly, liver involvement and cutaneous eruption account for the commonest signs observed in affected patients. The course of the disease is classically divided into a systemic and a chronic-articular subtype and can be complicated by the onset of macrophage activation syndrome (MAS), characterized by excessive activation of macrophages and T-lymphocytes, with overwhelming systemic inflammation and potentially fatal multi-organ failure [2, 3]. Other potentially life-threatening complications encompass acute respiratory distress syndrome (ARDS), fulminant hepatitis and cardiovascular complications such as myocarditis and pericarditis [4].

The hallmark cutaneous manifestation of Still’s disease is a transient, salmon-coloured maculopapular rash that typically occurs during febrile episodes [5]. The appearance of the rash can be fleeting, frequently resolving within a few hours, which can pose challenges for clinical observation. In addition to the classic salmon-coloured rash, patients with Still’s disease may present with other atypical skin manifestations, the most common being persistent pruritic papules and plaques (PPPP) [6]. The occurrence of atypical skin lesions is well documented in adult-onset Stills disease (AOSD) and has been occasionally described in systemic juvenile idiopathic arthritis (sJIA) [7]. Given the plethora of skin manifestations in Still’s disease, recognizing them is crucial for dermatologists to achieve an accurate diagnosis and effective management, as atypical skin lesions can often lead to misdiagnosis. Indeed, due to the rarity of the disease, large observational studies on a wide number of patients with Still’s disease, particularly focusing on skin manifestations, are lacking. The aim of this study was to thoroughly explore a large international cohort of patients with Still’s disease, with a focus on skin lesions, and to correlate these with patients’ ethnicities, age at onset, disease course and severity, as well as with the onset of specific clinical complications.

## Methods

### Study design and participants

This study was based on data from the International AutoInflammatory Disease Alliance (AIDA) Network Registry dedicated to Still’s disease [8]. Data were retrospectively collected. The enrolment period started in July 2021; we retrospectively extrapolated information on 518 Still’s disease patients up to January 2024. Still’s disease was classified according to internationally accepted criteria proposed by Yamaguchi *et al.* and/or Fautrel *et al.* for adult patients [9, 10]; the International League of Associations for Rheumatology (ILAR) and/or Pediatric Rheumatology International Trials Organization (PRINTO) criteria were employed for patients aged <16 years [11, 12].

Age, sex, ethnic origin, age at disease diagnosis and onset, clinical features, and patterns of the disease, cutaneous manifestations, the occurrence of life-threatening complications such as MAS were collected. Three clinical patterns (monocyclic, polycyclic and chronic) of the disease course were considered. A monocyclic course was defined as a single episode lasting >2 months but <1 year, followed by sustained remission through the entire follow-up period. A polycyclic course was characterized by recurrent systemic flares with remission between inflammatory episodes. A chronic course was defined as a disease pattern characterized by persistent disease activity with prominent joint involvement and less intense systemic inflammatory manifestations [13]. The MAS was defined according to either the 2016 classification criteria proposed by Ravelli *et al.* [14], and/or the HLH-2004 criteria developed by the Histiocyte Society [15], and/or the HScore, which has been developed and validated for the diagnosis of reactive HLH in both rheumatologic and non-rheumatologic conditions [15].

Cutaneous manifestations of the disease were documented, including the characteristic salmon-coloured skin rash, PPPP as the most frequent atypical presentation of Still’s disease, and a variety of less common atypical skin lesions, ranging from flagellate erythema to vesiculopustular eruptions. Moreover, the presence of pruritus as a symptom was assessed. Additional clinical characteristics recorded included: fever, sore throat, arthralgia or arthritis, myalgia, lymphadenopathy, splenomegaly, hepatomegaly, or liver dysfunction, abdominal or thoracic pain, pleuritis, pericarditis and ocular, renal, neurological and cardiac involvement. Skin lesions were considered as atypical cutaneous manifestations of Still’s disease if occurring during active phases of the disease, characterized by the presence of at least fever, hepatosplenomegaly, lymphadenopathy or severe joint involvement, accompanied by markedly increased ESR, CRP and ferritin levels. Alternatively, skin manifestations were considered atypical if histopathological examination demonstrated features consistent with predominant neutrophilic infiltration. The severity of the disease was assessed using the Pouchot score, with a threshold of 7 [16, 17]. It assigns points to fever, rash, pleuritis, pneumonia, pericarditis, hepatomegaly or abnormal liver function tests, splenomegaly, lymphadenopathy, leukocytes ≥15 000 mm^3^, sore throat, myalgia and abdominal pain as key clinical and laboratory findings, with each item worth 1 point. Conversely, the Rau score, also known as modified Pouchot score, has been proposed and employed to assess disease activity, with a threshold of 4 between active and inactive disease [18]. It focuses on a slightly different range of systemic features and inflammatory parameters, as it incorporates serum ferritin levels ≥3000 µg/l and arthritis, replacing splenomegaly and abdominal pain. The term ‘liver involvement’ was defined as the presence of abnormal liver function and/or hepatomegaly, as identified by US and/or other radiological documentation. Fulminant hepatitis was defined as the rapid onset of acute liver failure characterized by severe hepatic dysfunction, coagulopathy and hepatic encephalopathy in a patient with no pre-existing liver disease [19].

The aim of the study was to investigate cutaneous manifestations in a large international cohort of patients with Still’s disease, evaluating their correlation with ethnic origin, age at disease onset, patterns of the disease, life-threatening complications such MAS, Pouchot score and modified Pouchot score.

### Protocol approval

The study was approved by the Ethics Committee of the University Hospital of Siena, Siena, Italy (Protocol Number 14951) as part of the AIDA Program. The study protocol conformed to the tenets of the Helsinki Declaration. Written informed consent to participate in the international AIDA Registry for Still’s disease patients was obtained from all patients and/or their legal guardians.

### Statistical analysis

Descriptive statistics included mean and standard deviation (S.D.) for continuous variables, while frequency and percentages were reported for qualitative variables. To determine whether the data distribution was normal or not, the Shapiro–Wilk test was employed. Chi-squared test, Fisher’s exact test and Student’s *t* test were performed to compare two groups. To evaluate the difference of quantitative variables between age ranges and ethnicities, ANOVA test was used. As *post hoc* analysis of ANOVA, the Tuckey procedure was assessed. Instead, multiple Fisher exact tests with false discovery rate correction were performed as *post hoc* of the chi-squared test. The *P*-values reported for the *post hoc* analysis have already been adjusted for multiple comparisons.

Effect sizes were calculated to complement statistical significance testing and to provide an estimate of the strength of associations. For categorical variables, Cramer’s V was used, while for continuous variables analysed via ANOVA, Eta-squared was reported. Both statistics range from 0 to 1, with values closer to 1 indicating stronger associations and 0 representing no association. The interpretation of Cramer’s V depends on the size of the contingency table: for 2 × 2 or 3 × 2 contingency tables, values of 0.30 or higher are indicative of a strong association, while larger tables require higher thresholds (≥ 0.50 for 4 × 2 tables) to denote strong associations. Regarding Eta-squared, values ≥0.14 were interpreted as indicative of a strong difference.

A *P* < 0.05 was considered statistically significant. All data were assessed using software version R 4.1.0 (RStudio Team, 2023; Boston, MA, USA: Posit Software, PBC).

## Results

### Distribution of Still’s disease manifestations

A total of 518 patients (41.3% males) were included in the present study. Demographic data and manifestations referring to Still’s disease onset, including cutaneous manifestations, are summarized in Table 1. The frequency of extracutaneous signs observed up to the time of the enrolment is listed in Supplementary Table S1.

**Table 1.** Demographic and clinical manifestations, including skin lesions, of the study population

	*N* (%) 518
Male sex	213 (41.3)
Ethnic origin	
Arab	50 (9.9)
Asian	6 (1.2)
Black	7 (1.4)
Hispanic	45 (8.9)
Jewish	1 (0.2)
Native American	1 (0.2)
White	390 (77.5)
Other	3 (0.6)
Age at disease onset (years), mean (S.D.)	31.93 (17.39)
Type of Still’s disease	
Chronic-articular	101 (21.6)
Monocyclic	162 (34.7)
Polycyclic	137 (29.3)
Undefined course	67 (14.3)
Higher body temperature reached during the first episode (°C) (mean (S.D.))	39.35 (2.01)
Skin manifestations *N*, (%)	
Salmon-coloured evanescent skin rash	304 (63.9)
Pruritus associated to skin lesions	104 (29.6)
Other skin manifestations	122 (25.8)
Macules	40 (7.7)
Erythema	27 (5.2)
Persistent pruritic papules and/or plaques (PPPP)	25 (4.8)
Urticarial eruptions	31 (5.9)
Pustular lesions on the extremities and the trunk	4 (0.8)
Lichenoid papules	3 (0.6)
Alopecia	3 (0.6)
Pigmented plaques	3 (0.6)
Eczema-like lesions	2 (0.4)
Prominent linear dermographism‐like lesions	2 (0.4)
Vesiculopustular eruptions	2 (0.4)
Acneiform lesions	2 (0.4)
Erythematous maculopapules mimicking Gottron papules	2 (0.4)
Insect bite-like papulonodules	1 (0.2)
Erythema chronicum migrans	1 (0.2)
Erysipelas-like	1 (0.2)
Oedema of the eyelids	1 (0.2)
Flagellate erythema-type manifestations	1 (0.2)
Prurigo pigmentosa-like rash	1 (0.2)

Overall, 390 (77.5%) patients were White, 50 (9.9%) Arabian, 45 (8.9%) Hispanic and 18 (3.4%) recorded as ‘others’ (including Asians, Black, Jewish, Native Americans). The mean age at disease onset was 31.93 ± 17.39 years. Regarding skin manifestations, 304 (63.9%) patients presented with a salmon-coloured evanescent skin rash. In addition, macules were the most common cutaneous findings in 40 (7.7%) patients, followed by urticarial eruptions (5.9%), erythema (5.2%) and PPPP (4.8%). Less common manifestations were represented by pigmented plaques (0.6%), erythematous maculopapular lesions mimicking Gottron papules (0.4%), prominent linear dermographism‐like lesions (0.4%), flagellate erythema (0.2%) and prurigo pigmentosa-like rash (0.2%). Finally, pruritus was associated with skin lesions in 29.6% of cases. Arthralgia (84.5%) and arthritis (57%), splenomegaly (36.1%) and liver involvement (35%), lymphadenopathy (50.2%) and sore throat (56.4%) represented the most common extracutaneous manifestations at disease onset. Overall, 101 (21.6%) patients presented with a chronic-articular pattern of Still’s disease, 162 (34.7%) showed a monocyclic course and 137 (29.3%) patients had a polycyclic one, while 67 (14.3%) were not classified.

### Distribution of skin manifestations among different patient groups

Considering age at disease presentation, we noted that the occurrence of a salmon-coloured evanescent skin rash was more common in the <16 age group (80.2%) compared with the 16–60 (60.2%) and >60 (62.2%) age groups; however, the statistical significance was achieved only comparing the <16 age group with the 16–60 group (*P*-adjusted = 0.002). With regard to pruritus, it was more frequently observed in the 16–60 (33.2%) and >60 (30.4%) age groups compared with the <16 group (14.7%), with statistical significance being observed in the 16–60 group vs the <16 group (*P*-adjusted = 0.008) (Table 2).

**Table 2.** Variations in the frequency of Still’s disease manifestations across different age groups

Variables	<16 years	16–60 years	>60 year	*P*-value	Effect size
*n*	*N* = 85	*N* = 366	*N* = 39		
Male, *n* (%)	41 (48.2)	144 (39.3)	16 (41.0)		0.068
Still subtype, *n* (%)					
Chronic-articular	21 (25.3)	67 (19.5)	12 (34.3)	0.089	0.102
Monocyclic	30 (36.1)	122 (35.6)	9 (25.7)	0.491	0.055
Polycyclic	22 (26.5)	107 (31.2)	6 (17.1)	0.182	0.086
Cutaneous Still manifestations					
Salmon-coloured evanescent skin rash, *n* (%)	65 (80.2)	212 (60.2)	23 (62.2)	0.003^a^	0.156
Atypical skin manifestations, *n* (%)	15 (18.5)	99 (28.4)	6 (17.1)	0.090	0.102
Macules, *n* (%)	5 (5.9)	34 (9.3)	1 (2.6)	0.242	0.076
PPPP, *n* (%)	2 (2.4)	22 (6.0)	1 (2.6)	0.291	0.071
Erythema, *n* (%)	1 (1.2)	24 (6.6)	2 (5.1)	0.146	0.089
Pruritus, *n* (%)	10 (14.7)	85 (33.2)	7 (30.4)	0.012^a^	0.160

The table also reports *P*-values and effect sizes calculated using Cramer’s V (for categorical variables) and Eta-squared (for continuous variables). Both statistics range from 0 (no association) to 1 (perfect association). Interpretation of Cramer’s V depends on the size of the contingency table; in this case, for a 3 × 2 table, values of 0.30 or higher are generally considered indicative of a strong association between different age groups and the observed variables. For continuous variables, Eta-squared values of 0.14 or higher reflect a strong difference. In order to account for multiple comparisons across the three age groups, the Tukey *post hoc* test following ANOVA or the false discovery rate correction following multiple Fisher’s exact tests was employed.

a <16 significantly different from 16 to 60.

PPPP, persistent pruritic papules and plaques.

**Figure 1.**
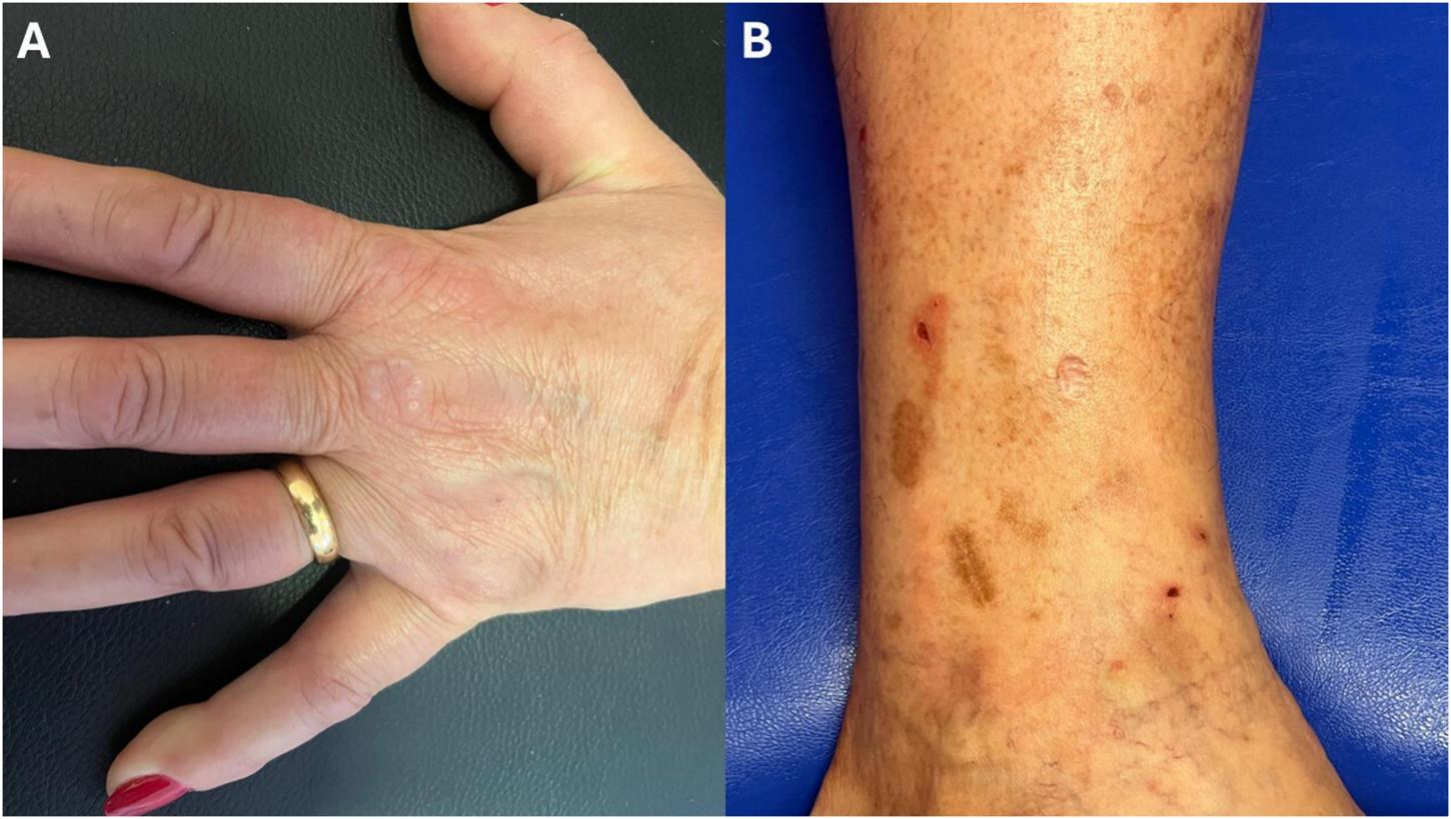
Two examples of atypical cutaneous manifestations of Still’s disease. (**A**) Slightly erythematous, annular plaque with a moderately firm, rope-like border and central clearing on the dorsal aspect of the third metacarpophalangeal joint of the left hand. Discrete 1–2 mm papules are noted at the periphery of the lesion. The lesion closely resembles the granuloma annulare. (**B**) Pigmented brown macules distributed on the dorsal aspect of the right leg. The lesions are flat, assume a linear morphology and are not pruritic

Regarding ethnicities, Hispanics showed a significantly higher rate of atypical skin manifestations (44.2%) compared with Arabs (18.4%, *P*-adjusted = 0.036) and White (24.4%, *P*-adjusted = 0.036). Also, Hispanic patients showed a significantly higher prevalence of macules (20.0%) compared with White (6.4%, *P*-adjusted = 0.027). Consistently, PPPP were significantly more common in Hispanics (17.8%) and in the ‘other’ ethnicity group (22.2%) vs Arabs (2%) and White (2%) (Hispanic vs Arabs: *P*-adjusted = 0.023; ‘other’ group vs Arabs: *P*-adjusted = 0.023; Hispanic vs White: *P*-adjusted = 0.002; ‘other’ group vs White: *P*-adjusted = 0.011). Finally, a higher percentage of Hispanic patients (56.2%) reported pruritus associated with skin lesions compared with Arabs (7.7%, *P*-adjusted < 0.001) and White (29.3%, *P*-adjusted = 0.008). Moreover, the frequency of pruritus among Arabs was significantly lower compared with White (*P*-adjusted = 0.008) and the ‘other’ ethnicity group (38.5%, *P*-adjusted = 0.026) (Table 3). Figure 1 shows two examples of atypical cutaneous manifestations of Still’s disease.

**Table 3.** Variations in the frequency of Still’s disease manifestations across different ethnicities

Variables	Arab	White	Hispanic	Other^a^	*P*-value	Effect size
*n*	50	390	45	18		
Male, *n* (%)	15 (30.0)	171 (44.0)	20 (44.4)	3 (16.7)	0.038	0.129
Age at disease onset, mean (S.D.), years	28.11 (12.20)	31.98 (18.58)	34.55 (10.83)	32.11 (16.33)	0.334	0.007
Still subtype, *n* (%)						
Chronic-articular	10 (20.8)	81 (23.4)	2 (4.7)	6 (33.3)	0.025^b,^^c^	0.144
Monocyclic	25 (52.1)	113 (32.7)	15 (34.9)	4 (22.2)	0.040	0.135
Polycyclic	3 (6.2)	102 (29.5)	23 (53.5)	5 (27.8)	<0.001^b^^,^^d^^,^^e^^,^^f^	0.232
Cutaneous Still manifestations						
Salmon-coloured evanescent skin rash, *n* (%)	37 (77.1)	224 (63.5)	22 (50.0)	10 (58.8)	0.059	0.127
Atypical skin manifestations = yes (%)	9 (18.4)	86 (24.4)	19 (44.2)	6 (33.3)	0.020^b^^,^^e^	0.146
Macules, *n* (%)	3 (6.0)	25 (6.4)	9 (20.0)	3 (16.7)	0.006^b^	0.156
PPPP, *n* (%)	1 (2.0)	12 (3.1)	8 (17.8)	4 (22.2)	<0.001^b^^,^^e^^,^^f^^,^^g^	0.248
Erythema, *n* (%)	2 (4.0)	22 (5.6)	3 (6.7)	–	0.703	0.053
Pruritus, *n* (%)	3 (7.7)	76 (29.3)	18 (56.2)	5 (38.5)	<0.001^b^^,^^d^^,^^e^^,^^f^	0.243

The table also reports *P*-values and effect sizes calculated using Cramer’s V (for categorical variables) and Eta-squared (for continuous variables). Both statistics range from 0 (no association) to 1 (perfect association). Interpretation of Cramer’s V depends on the size of the contingency table; in this case, for a 4 × 2 table, values of 0.50 or higher are generally considered indicative of a strong association between ethnic groups and the observed variables. For continuous variables, Eta-squared values of 0.14 or higher reflect a strong difference. In order to account for multiple comparisons across the four ethnic groups, the Tukey *post hoc* test following ANOVA or the false discovery rate correction following multiple Fisher’s exact tests was employed.

a Including Asians, Black, Jewish, Native Americans.

b White significantly different from Hispanic.

c Hispanic significantly different from ‘Other’.

d Arab significantly different from White.

e Arab significantly different from Hispanic.

f Arab significantly different from ‘Other’.

g White significantly different from ‘Other’.

PPPP, persistent pruritic papules and plaques.

When analysing disease patterns, no specific cutaneous manifestation of Still’s disease was significantly different in any disease course. The frequency of atypical cutaneous manifestations was higher in the chronic-articular pattern (32.3%) compared with the monocyclic (23.2%) and polycyclic (26.1%) patterns, but statistical significance was not achieved (*P* = 0.275). Moreover, pruritus was observed more frequently in the polycyclic group (36.9%) than in the chronic-articular (33.8%) and monocyclic (21.6%) groups, with a statistically significant difference between polycyclic vs monocyclic (*P*-adjusted = 0.049) groups (Supplementary Table S2).

Demographic, clinical and cutaneous manifestations of Still’s disease contributing to the Pouchot score at baseline are listed in Table 4. A significantly higher percentage of patients with Pouchot score ≥7 during the first febrile episode had a salmon-coloured evanescent skin rash (84.2%) compared with those with Pouchot score <7 (58.8%) (*P* < 0.001). This finding was still significant when the Rau score was considered (*P* < 0.05) (Supplementary Table S3).

**Table 4.** Variations in the frequency of Still’s disease manifestations according to disease severity measured in terms of Pouchot score

Variables	Pouchot < 7	Pouchot ≥ 7	*P*-value	Effect size
*n*	340	96		
Male, *n* (%)	136 (40.0)	39 (40.6)	1.000	0.005
Age at disease onset, mean (S.D.), years	32.14 (17.32)	32.57 (17.94)	0.831	0.001
Still subtype, *n* (%)				
Chronic-articular	78 (24.1)	15 (16.0)	0.127	0.071
Monocyclic	111 (34.3)	32 (34.0)	1.000	0.002
Polycyclic	87 (26.9)	32 (34.0)	0.219	0.067
Cutaneous Still manifestations				
Salmon-coloured evanescent skin rash, *n* (%)	198 (58.8)	80 (84.2)	<0.001	0.220
Atypical skin manifestations, *n* (%)	87 (26.0)	23 (24.5)	0.872	0.014
Macules, *n* (%)	30 (8.8)	10 (10.4)	0.782	0.023
PPPP, *n* (%)	18 (5.3)	6 (6.2)	0.913	0.017
Erythema, *n* (%)	20 (5.9)	4 (4.2)	0.691	0.031
Pruritus, *n* (%)	71 (29.7)	23 (28.0)	0.885	0.016

The table also reports the *P*-value and the effect sizes through Cramer’s V (for categorical variables) and Eta-squared (for continuous variables). Both statistics range from 0 (no association) to 1 (perfect association). Values of Cramer’s V ≥ 0.30 or Eta-squared ≥ 0.14 are considered to reflect a strong association or a strong difference, respectively, between the different thresholds of the Pouchot score and the observed variables.

PPPP, persistent pruritic papules and plaques.

No statistically significant association between the occurrence of MAS and the presence of salmon-coloured evanescent skin rash or other skin manifestations was observed (*P* > 0.05) (Supplementary Table S4).

## Discussion

Recognizing Still’s disease based on its cutaneous manifestations remains critical among dermatologists, especially due to the wide range of different atypical skin manifestations. This lack of familiarity can result in diagnostic delays, which may contribute to potential complications. Early detection is essential for timely intervention, as it helps prevent the progression of systemic involvement and reduces the risk of severe outcomes. Based on current evidence, AOSD might be characterized by a wide range of dermatological manifestations, including salmon-coloured rash, PPPP, and various other eruptions such as prurigo pigmentosa-like lesions, vesiculopustular lesions or flagellate erythema [20]. In contrast, the skin manifestations in sJIA are generally more uniform, often limited to the characteristic evanescent salmon-coloured rash, and less varied in terms of morphology and distribution [21]. For this reason, only the typical rash of Still’s disease has been attributed to this disorder for a long timeframe, with atypical presentations misleading clinicians when facing with the diagnosis.

To date, some studies in the literature have explored the cutaneous manifestations of Still’s disease and their correlation with other clinical features. However, these studies have generally been conducted on relatively small patient cohorts. In this perspective, Ruscitti *et al.* analysed a cohort of 100 AOSD patients, correlating various clinical manifestations to disease progression patterns (monocyclic, polycyclic and chronic). The typical skin rash was found to be more frequent in patients with a ‘monocyclic’ and ‘chronic-articular’ course, even though a statistical significance was not achieved [16].

Regarding any association between skin rash and the development of MAS, the results observed in the present study are in line with what observed in a further study on 119 patients by Ruscitti *et al.* [22]. In particular, no association was found between the typical skin rash and the occurrence of MAS. However, while Ruscitti and colleagues only addressed cutaneous manifestations in terms of ‘skin rash’, referring to the typical one, this current study explores the full spectrum of AOSD skin lesions, confirming a lack of association with MAS.

At current, only a few studies have specifically focused on the various types of cutaneous lesions possibly associated with AOSD. In detail, Sato *et al.* analysed typical and atypical skin lesions and their frequency in a cohort of AOSD 28 patients [21]. They correlated the various types of lesions to the age at disease onset, distinguishing between patients younger and older than 65 years. As in our study, they found a significantly higher prevalence of typical rash in younger patients compared with older ones. Conversely, among the atypical skin rashes, PPPP was more common in patients over 65 years old. However, this last finding was not confirmed in our study, where none of the atypical skin lesions showed a significant association with the age of AOSD onset.

In the study by Narváez Garcia *et al.*, typical and atypical cutaneous manifestations were analysed in 81 patients deriving from case reports and series, evaluating their impact on prognosis and mortality of AOSD. They observed that the development of atypical rash was associated with a worse prognosis due to a higher mortality rate compared with patients with typical rash, as well as with a Pouchot score ≥7 [23]. Nevertheless, our study did not confirm this finding. Indeed, typical rash was significantly associated with greater disease severity, according to the Pouchot score. Conversely, no statistically significant association was found between atypical cutaneous manifestations and higher or lower severity scores, whether according to Pouchot or Rau scores.

A further study from Eveillard *et al.* investigated the spectrum of skin involvement of sJIA. These authors found that sJIA patients with atypical skin presentations were more likely to experience long-term, persistent disease, requiring more intensive or prolonged treatments [7].

Interestingly, no studies have explored the cutaneous manifestations of Still’s disease across different ethnic groups so far. This represents a significant gap in our understanding of the disease and investigating these variations could provide valuable insights into the Still’s disease’s behaviour and progression in diverse ethnic backgrounds, potentially leading to more tailored and effective diagnostic and treatment approaches. In our study, no statistically significant differences were found in the frequency of typical rash across different ethnic groups. However, atypical cutaneous variants were significantly more frequent among Hispanics compared with other White and Arabs. When analysing specific atypical cutaneous eruptions, it was observed that macules were significantly more frequent in Hispanics compared with other White. Furthermore, PPPP were significantly more prevalent in the Hispanic and ‘other’ ethnic groups, in comparison to Arabs or White. Noteworthy, pruritus was significantly more commonly reported among Hispanics than in Arabs or White. It is known that the clinical presentation of chronic inflammatory skin diseases, such as atopic dermatitis, can vary significantly among ethnic groups due to genetic, environmental and cultural factors, as well as differing immunological pathways [24]. It is plausible that this concept also applies to Still’s disease, where the heterogeneity of cutaneous manifestations across different ethnicities may reflect distinct immunopathogenic mechanisms driven by diverse genetic backgrounds. In this context, both HLA and non-HLA genes, as well as various immune signalling pathways, are known to play a role in the pathogenesis of the disease [25]. Although to the best of our knowledge ethnicity-specific genetic studies have not yet been conducted in patients with Still’s disease, evidence from other immune-mediated disorders indicates that allele frequencies and genetic susceptibility factors can differ significantly among populations [26]. These variations may contribute to differential immune responses, potentially accounting for the observed diversity in cutaneous involvement. However, specific genetic studies are needed to explore and eventually confirm this hypothesis.

The main strength of this work relies on the large cohort of Still patients retrieved from an international registry. Nonetheless, some limitations need to be recognized. Firstly, the retrospective nature of the study accounts for some missing data, along with its inherent limitations. Secondly, a comparison between scientific studies on Still’s disease was complicated by the heterogeneity of methods employed in the literature and the lack of a consistent body of evidence on cutaneous manifestations of Still’s disease.

## Conclusion

In conclusion, various studies have analysed the different patterns of Still’s disease, clinical manifestations, the development of complications, as well as the age at disease onset, correlating these factors with patient prognosis so far. However, very few studies explored the wide range of cutaneous manifestations of Still’s disease, dissecting them in typical and atypical and correlating them to specific patient and disease characteristics.

Comprehensively, the present study enhances dermatologists’ awareness of the diverse cutaneous lesions that may represent heterogeneous manifestations of Still’s disease.

## Supplementary Material

keaf512_Supplementary_Data

## Data Availability

The raw data supporting the conclusions of this article will be made available by the authors, without undue reservation. Requests to access these datasets should be directed to the corresponding author: Luca Cantarini, MD, PhD, Research Center of Systemic Autoinflammatory Diseases and Behçet’s Disease Clinics, Department of Medical Sciences, Surgery and Neurosciences, University of Siena. cantariniluca@hotmail.com.
