# Supplementary material for: Unfolding dermatological spectrum of Still’s disease: a cohort study from the International AIDA Network Still’s Disease Registry
Source: Rheumatology (Oxford). 2025 Sep 30;65(1):keaf512. doi: 10.1093/rheumatology/keaf512 (PMC12862393; doi:10.1093/rheumatology/keaf512)
Supplement: keaf512_Supplementary_Data [file keaf512_supplementary_data.docx]

**Supplementary Table S1. Extracutaneous manifestations of the study population**

| **Extracutaneous manifestation** | N, (%)  518 |
| --- | --- |
| Arthralgia | 399 (84.5) |
| Arthritis | 259 (57.0) |
| *Type of arthritis* |  |
| Monoarthritis (1 joint involved) | 15 (6.3) |
| Oligoarthritis (2-4 joints involved) | 98 (41.2) |
| Polyarthritis (5 or more joints involved) | 125 (52.5) |
| Pharyngodynia | 264 (56.4) |
| Myalgia | 249 (53.2) |
| Lymphadenopathy | 237 (50.2) |
| Splenomegaly | 171 (36.1) |
| Liver involvement | 165 (35.0) |
| Hepatomegaly | 126 (24.3) |
| Abdominal pain not associated to peritonitis | 66 (14.1) |
| Pleuritis | 63 (13.4) |
| Pericarditis | 58 (12.4) |
| Thoracic pain not associated to serositis | 50 (10.7) |
| Macrophage activation syndrome (MAS) | 44 (9.5) |
| Vomiting | 42 (8.9) |
| Diarrhea | 42 (8.9) |
| Acute hepatitis | 29 (5.6) |
| Abdominal effusions | 21 (4.5) |
| Other liver involvement | 20 (3.9) |
| Conjunctivitis | 17 (3.7) |
| Neurological involvement | 14 (3.0) |
| Cardiac complications | 13 (2.8) |
| Kidney involvement | 11 (2.4) |
| Acute respiratory distress syndrome (ARDS) | 7 (1.5) |
| Hepatic failure | 8 (1.5) |
| Peritonitis | 4 (0.9) |
| Uveitis | 3 (0.6) |
| Fulminant hepatitis | 3 (0.7) |
| Inflammatory orbital pseudotumor | 3 (0.6) |
| Scleritis | 2 (0.4) |
| Orchitis | 2 (0.4) |
| Pseudo-angiocholitis | 1 (0.2) |
| Jaundice | 1 (0.2) |

**Supplementary Table S2. Variations in the Frequency of Still's Disease Manifestations Across Disease Patterns. The table also reports the p-value and the effect sizes through Cramer’s V (for categorical variables) and Eta-squared (for continuous variables). Both statistics range from 0 (no association) to 1 (perfect association). For 3×2 contingency tables, a value of Cramer’s V ≥ 0.30 is considered to reflect at least a moderate association between the different Still’s disease courses and the observed variables. Values of Eta-squared ≥ 0.14 are interpreted as a strong difference for continuous variables. To account for multiple comparisons across the three different disease courses, the Tukey procedure was used for post hoc analysis following ANOVA, and the false discovery rate (FDR) correction was applied following multiple Fisher's exact tests.**

| Variables | Chronic-articular | Monocyclic | Polycyclic | p-value | Effect size |
| --- | --- | --- | --- | --- | --- |
| n | 101 | 162 | 137 |  |  |
| Male, n (%) | 41 (40.6) | 63 (38.9) | 60 (43.8) | 0.688 | 0.043 |
| Age at disease onset (years) (mean (SD)) | 33.95 (19.60) | 31.18 (17.11) | 29.79 (15.34) | 0.184 | 0.009 |
| **Cutaneous Still manifestations** | | | | | |
| Salmon-colored evanescent skin rash, n (%) | 60 (60.6) | 102 (64.6) | 86 (64.2) | 0.795 | 0.034 |
| Atypical skin manifestations, n (%) | 32 (32.3) | 36 (23.2) | 35 (26.1) | 0.275 | 0.082 |
| Macules, n (%) | 9 ( 8.9) | 14 ( 8.6) | 12 ( 8.8) | 0.997 | 0.004 |
| PPPP, n (%) | 10 ( 9.9) | 5 ( 3.1) | 9 ( 6.6) | 0.073 | 0.114 |
| Erythema, n (%) | 9 ( 8.9) | 9 ( 5.6) | 7 ( 5.1) | 0.437 | 0.064 |
| Pruritus, n (%) | 25 (33.8) | 25 (21.6) | 38 (36.9) | 0.034^a^ | 0.152 |

**Legend:** SD, Standard deviation; PPPP, persistent pruritic papules and plaques.

^a^Monocyclic significantly different from Polycyclic

**Supplementary Table S3. Variations in the Frequency of Still's Disease Manifestations according to disease severity measured in terms of modified Pouchot score (Rau score). The table also reports the p-value and the effect sizes through Cramer’s V (for categorical variables) and Eta-squared (for continuous variables). Both statistics range from 0 (no association) to 1 (perfect association). Values of Cramer’s V ≥ 0.30 or Eta-squared ≥ 0.14 are considered to reflect a strong association between the different thresholds of the modified Pouchot score and the observed variables.**

| Variables | **Modified Pouchot<7** | **Modified Pouchot≥7** | **p-value** | **Effect size** |
| --- | --- | --- | --- | --- |
| n | 307 | 121 | 307 |  |
| Male, n (%) | 128 (41.7) | 45 (37.2) | 0.456 | 0.041 |
| Age at disease onset (years) (mean (SD)) | 31.37 (17.85) | 34.24 (16.53) | 0.128 | 0.005 |
| Still subtype, n (%) | | | | |
| Chronic-articular | 67 (22.9) | 24 (20.2) | 0.628 | 0.030 |
| Monocyclic | 102 (34.9) | 40 (33.6) | 0.888 | 0.013 |
| Polycyclic | 80 (27.4) | 36 (30.3) | 0.644 | 0.029 |
| **Cutaneous Still manifestations** | | | |  |
| Salmon-colored evanescent skin rash, n(%) | 177 (58.2) | 97 (80.8) | <0.001 | 0.213 |
| Atypical skin manifestations, n (%) | 80 (26.4) | 28 (23.7) | 0.660 | 0.027 |
| Macules, n (%) | 28 (9.1) | 12 (9.9) | 0.944 | 0.012 |
| PPPP, n (%) | 15 (4.9) | 9 (7.4) | 0.424 | 0.050 |
| Erythema, n (%) | 19 (6.2) | 5 (4.1) | 0.549 | 0.040 |
| Pruritus, n (%) | 64 (30.2) | 29 (27.9) | 0.771 | 0.024 |

**Legend:** SD, Standard deviation; PPPP, persistent pruritic papules and plaques

**Supplementary Table S4. Variations in the Frequency of Still's Disease Manifestations according to the occurrence of MAS.** **The table also reports the p-value and the effect sizes through Cramer’s V (for categorical variables) and Eta-squared (for continuous variables). Both statistics range from 0 (no association) to 1 (perfect association). Values of Cramer’s V ≥ 0.30 or Eta-squared ≥ 0.14 are considered to reflect a strong association between the MAS occurrence and the observed variables.**

| **Variables** | **No MAS** | **MAS** | **p-value** | **Effect Size** |
| --- | --- | --- | --- | --- |
| N | 420 | 44 |  |  |
| Male, n (%) | 174 (41.4) | 16 (36.4) | 0.625 | 0.030 |
| Age at disease onset (years) (mean (SD)) | 32.44 (17.05) | 26.31 (18.31) | 0.026 | 0.01 |
| Still subtype, n (%) | | | |  |
| Chronic-articular | 96 (23.7) | 3 (7.1) | 0.024 | 0.116 |
| Monocyclic | 133 (32.8) | 20 (47.6) | 0.080 | 0.091 |
| Polycyclic | 121 (29.9) | 11 (26.2) | 0.748 | 0.024 |
| **Cutaneous Still manifestations** | | | |  |
| Salmon-colored evanescent skin rash, n (%) | 261 (62.9) | 30 (69.8) | 0.468 | 0.042 |
| Atypical skin manifestations, n (%) | 109 (26.3) | 10 (23.8) | 0.872 | 0.016 |
| Macules, n (%) | 37 (8.8) | 3 (6.8) | 0.869 | 0.21 |
| PPPP, n (%) | 22 (5.2) | 3 (6.8) | 0.928 | 0.021 |
| Erythema, n (%) | 25 (6.0) | 1 (2.3) | 0.506 | 0.047 |
| Pruritus, n (%) | 88 (28.8) | 12 (35.3) | 0.552 | 0.043 |

**Legend**: MAS, macrophage activation syndrome; SD, Standard deviation; PPPP, persistent pruritic papules and plaques
